# Is CONUT score a prognostic index in patients with diffuse large cell lymphoma?

**DOI:** 10.3906/sag-2101-406

**Published:** 2021-08-30

**Authors:** Gülsüm AKGÜN ÇAĞLIYAN, Sibel HACIOĞLU, Başak ÜNVER KOLUMAN, Kadir İLKKILIÇ, Rukiye NAR, Mehmet Nuri BAŞER, Aslı BOZDEMİR, Hande ŞENOL, Nilay ŞEN TÜRK, Veysel EROL, Onurcan YILDIRIM, Ömer ÇAĞLIYAN, Nil GÜLER

**Affiliations:** 1 Department of Hematology, Faculty of Medicine, Pamukkale University, Denizli Turkey; 2 Department of Hematology, Recep Tayyip Erdogan University Traning and Research Hospital, Rize Turkey; 3 Department of Biochemistry, Faculty of Medicine, Pamukkale University, Denizli Turkey; 4 Department of Internal Medicine, Faculty of Medicine, Pamukkale University, Denizli Turkey; 5 Department of Biostatistics, Faculty of Medicine, Pamukkale University, Denizli Turkey; 6 Department of Pathology, Faculty of Medicine, Pamukkale University, Denizli Turkey; 7 Department of Internal Medicine, Şemdinli State Hospital, Hakkari Turkey; 8 Department of Cardiology, Denizli State Hospital, Denizli Turkey

**Keywords:** Lymphoma, Controlling Nutritional Status score, survival, prognosis

## Abstract

**Background/aim:**

The aim of the study was to evaluate the effect of Controlling Nutritional Status (CONUT) score on the prognosis in patients with diffuse large B-cell lymphoma (DLBCL).

**Materials and methods:**

The present study was a retrospective study. The CONUT score was calculated based on serum albumin, total cholesterol and lymphocyte levels. This study included a total of 266 patients, 131 (49.2%) were female and 135 (50.8%) were male. The median follow-up period was 51 months (range: 1–190).

**Results:**

The median age was 64 years. The cut off CONUT was 1.5. There was a significant difference between patients with high (≥ 2) or low (< 2) CONUT scores in terms of overall survival (OS) and progression-free survival (PFS). The 5-year OS and PFS in patients with high CONUT score was 52.1% and 49.7%. The 5-year OS and PFS in patients with low CONUT score was 79.8% and 75.6% (p < 0.001). In the multivariate analysis for OS, age ≥ 65 years (HR = 1.80, p = 0.028), Eastern Cooperative Oncology Group (ECOG) > 1 (HR = 2.04, p = 0.006), stage IIIA–IVB disease (HR = 2.75, p = 0.001) and the CONUT score (HR = 1.15, p = 0.003) were found statistically significant. In the multivariate analysis for PFS, age ≥ 65 years (HR = 2.02, p = 0.007), stage IIIA–IVB disease (HR = 2.42, p = 0.002) and the CONUT score (HR = 1.19, p = 0.001) were found to be significant parameters.

**Conclusion:**

High CONUT score reduces OS and PFS in DLBCL. CONUT score is an independent, strong prognostic index in patients with DLBCL.

## 1. Introduction

Diffuse large B-cell lymphoma (DLBCL) is the most common Non-Hodgkin lymphoma (NHL), constituting approximately 30%–40% of all NHL patients. Although it is an aggressive tumour, 60%–70% of the patients are cured with standard rituximab, cyclophosphamide, hydroxydaunorubicin, vincristine and prednisone (R-CHOP) chemotherapy [1]. However, approximately one-third of the patients are refractory to standard R-CHOP therapy. Gene expression profile and International Prognostic Index (IPI) are useful parameters in identifying high-risk patients [2]. The relationship between prognostic nutritional index and prognosis has been shown in patients with DLBCL [3]. 

The Controlling Nutritional Status (CONUT) score is a significant indicator used to identify patients with malnutrition in recent years. This score is calculated based on serum albumin, total cholesterol and lymphocyte counts. Serum albumin, total cholesterol and lymphocyte counts indicate protein reserve, calorie status and immune function, respectively. It is known that high CONUT score has an effect on the prognosis in patients who have undergone gastrointestinal surgery, cardiovascular disease, end-stage renal disease and malignant tumours [4-7]. In our study, we evaluated the survival and prognostic impact of the CONUT score in patients with DLBCL. The aim of the study was to evaluate the effect of Controlling Nutritional Status (CONUT) score on the prognosis in patients with diffuse large B-cell lymphoma (DLBCL).

## 2. Materials and methods

### 2.1. Patients

The present study included 266 DLBCL patients who were followed between 2012 and 2020 in the Department of Hematology, Faculty of Medicine, Pamukkale University. The study cohort was retrospectively enrolled. The median follow-up period was 51 months (range: 1–190). The final follow-up date was May 2020. The present study was approved by the Ethics Committee of the Faculty of Medicine, Pamukkale University. No procedures were performed, and no interventions were made during the study because of the retrospective study design. Patients with primary central nervous system lymphoma, human immunodeficiency virus-associated lymphoma and only palliative treatment were excluded. Patients who receive lipid lowering therapy were excluded. As our centre is not performing allogeneic stem cell transplantation, patients who received allogeneic stem cells were excluded. Performance status (PS) was evaluated based on the Eastern Cooperative Oncology Group (ECOG) criteria. National Comprehensive Cancer Network International Prognostic Index (NCCN-IPI) was determined based on the age at the time of diagnosis, serum lactate dehydrogenase (LDH) levels, PS, stage and extranodal involvement. Normal range of LDH was 135–225 U/L. The values above 225 U/L were considered high. 

### 2.2. CONUT score 

The CONUT score was calculated based on serum albumin concentration, total lymphocyte count and total cholesterol levels. Albumin concentrations of **≥ **3.50 g/dL, 3.00–3.49 g/dL, 2.50–2.99 g/dL and < 2.50 g/dL were scored as 0, 2, 4 and 6 points, respectively. Total lymphocyte counts of ≥ 1600 mm^3^, 1200–1599 mm^3^, 800–1199 mm^3^ and < 800 mm^3^ were scored as 0, 1, 2 and 3 points, respectively. Total cholesterol levels of ≥ 180 mg/dL, 140–179 mg/dL, 100–139 mg/dL and < 100 mg/dL were scored as 0, 1, 2 and 3 points. The CONUT score was calculated based on the addition of points albumin, total lymphocyte and total cholesterol at the time of diagnosis. 

### 2.3. About chemotherapy 

R-CHOP (rituximab 375 mg/m^2^ on day 1, cyclophosphamide 750 mg/m^2^ on day 2, doxorubicin 50 mg/m^2^ on day 2, vincristine 1.4 mg/m^2 ^on day 2 and prednisone 100 mg/m^2^ on days 1–5) or R-mini-CHOP (at a 25% reduced dose) chemotherapy was given to patients with DLBCL every 21 days based on their age, PS and comorbidities. The median age of patients treated with R-mini-CHOP chemotherapy was 79 years (65–91 years). The pathological phenotype of patients treated with DA-REPOCH (rituximab 375 mg/m^2^ on day 1, etoposide 50 mg/m^2^ on days 1–4, doxorubicin 10 mg/m^2^ on days 1–4, vincristine 0.4 mg/m^2 ^on days 1–4, cyclophosphamide 750 mg/ m^2^ on day 5 and prednisone 100 mg/m^2^ on days 1–5) chemotherapy was non-germinal. DA-REPOCH chemotherapy regimen was repeated every 21 days. 

### 2.4. Statistical analyses

Overall survival (OS) was defined as the period between the time of diagnosis and the last follow-up or death. Progression-free survival (PFS) was defined as the period between the time of diagnosis and the last follow-up, progression, relapse or death. Normality was tested using the Shapiro–Wilk test. Mann–Whitney U test was used for nonparametric distribution comparison. Kruskal–Wallis variance test was used for comparing three different chemotherapy regimens. OS and PFS were predicted using the Kaplan–Meier method and were compared using the log-rank test. We performed univariate and multivariate analyses for OS and PFS using the COX regression model. The correlation between the CONUT score and OS and PFS was analysed using the Spearman test. The receiver operating characteristic (ROC) curve analysis revealed the distinctive cut-off value for CONUT. All data were analysed using the SPSS Statistics version 25.0 (IBM SPSS Statistics 25 software; IBM Corp., Armonk, NY, USA) A p value of <0.05 was considered statistically significant.

## 3. Results

### 3.1. Characteristics of patients

The present study included a total of 266 patients with DLBCL 131 (49.2%) females and 135 (50.8%) males. The median age was 64 years (range: 23–91). The median follow-up period was 51 months (range: 1–190). For initial therapy, 223 (83.8%), 12 (4.5%) and 31 (11.7%) patients received R-CHOP, R-CHOP-mini and DA-REPOCH therapy regimes, respectively. The pathological phenotype of patients treated with DA-REPOCH therapy was non-germinal (11.7%). The patients were evaluated based on the NCCN-IPI. The patients with low or low-intermediate risk were categorised as low-IPI and those with high-intermediate or high risk were categorised as high IPI. The median number of cycles of chemotherapy was 6 (range, 1–10). The demographic and laboratory data of the patients are presented in Table 1.

**Table 1:** Patients characteristics.

	median /(min-max)
Age (year)	64 (23– 91)
ECOG	0 (0–4)
White blood cell(x109/L)	7.78 (1.73–30.6)
Lymphocytes (x109/L)	1680 (430–5890)
Hemoglobin (gr/dL)	12.75 (6.4–16.9)
Platelet (x109/L)	269.5 (34–903)
AST ( IU/L)	19 (5–195)
ALT (IU/L)	17 (3–195)
Total bilirubin (mg/dL)	0.43(0.09–5.8)
Uric acid (mg/dL)	4.45(1.38–22)
Creatinine (mg/dL)	0.73(0.35–6.84)
LDH ( U/L)	213.5(114–3855)
Albumin (mg/dL)	4.18(1.72–5.15)
Total cholesterol (mg/dL)	190 (59–780)
CONUT score	1 (0–11)

ECOG: Eastern Cooperative Oncology GroupCONUT: Controlling Nutritional Status

We recorded remission, refractory and relapsed disease states from 266 patient files. Following the initial treatment, 168 (63.2%) patients were in remission. The disease progression was identified using positron emission tomography (PET-CT) or computed tomography (CT). Ninety-five patients (35.7%) died during the study. The median CONUT scores were 1 (range: 0–11). The median CONUT scores in patients with progressive disease were 2.5. The median CONUT scores in patients with no progressive disease were 1. The CONUT scores were higher in patients with progressive disease (p = 0.001). The comparison between the CONUT score and clinical characteristics and laboratory parameters was shown in Table 2.

**Table 2:** The comparation between the CONUT score and patients’ characteristics and laboratory parameters.

	CONUT score	p
	Median (min–max)	
Age <65	1 (0–9)	p = 0.001
Age≥65	2 (0–11)	
Sex (female)	1 (0–11)	p = 0.667
Sex (male)	1 (0–10)	
ECOG<1	1 (0–8)	p = 0.001
ECOG≥1	2 (0–11)	
LDH normal	1 (0–10)	p = 0.001
LDH>normal	2 (0–11)	
Bone marrow involvement (-)	1 (0–11)	p = 0.001
Bone marrow involvement (+)	3 (0–7)	
Extranodal disease (-)	1 (0–9)	p = 0.001
Extranodal disease (+)	2 (0–11)	
Stage (IA-IIB)	0 (0–11)	p = 0.001
Stage (IIIA-IVB)	2 (0–9)	
IPI (low,low intermediate)	0 (0–8)	p = 0.001
IPI (high intermediate,high)	3 (0–11)	
Progressive disease (-)	1 (0–9)	p = 0.001
Progressive disease (+)	2.5 (0–11)	
RCHOP chemotherapy	1 (0–11)	p = 0.153
RCHOP-mini chemotherapy	1 (0–10)	
DA REPOCH chemotherapy	2 (0–9)	

There was a statistically significant difference between the CONUT scores and prognostic factors. (Such as age, ECOG, clinical stage, LDH level, extranodal disease, bone marrow involvement, NCCN-IPI and progressive disease (Table 2). There was no significant difference between the patients’ sex and first-line chemotherapy and the CONUT scores. 

**Figure 1:**
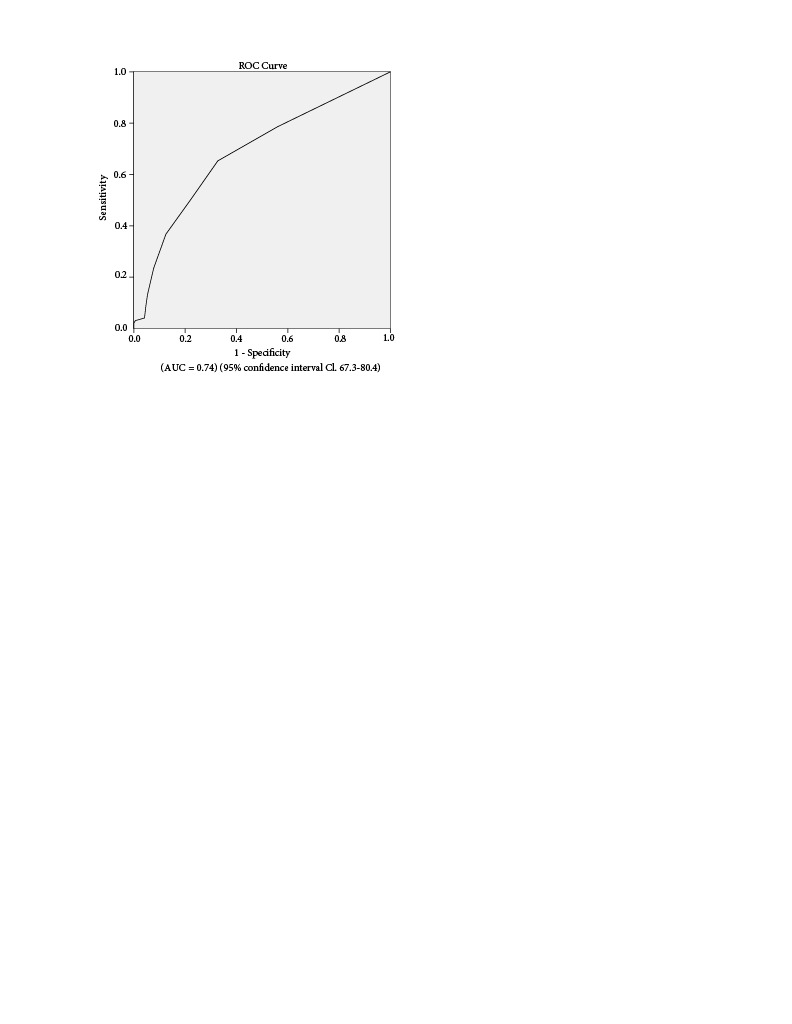
The CONUT score by ROC analysis.

The (ROC) curve analysis found the distinctive cut-off value for CONUT score to be 1.5 (The state of alive or censored from diagnosis and died within from diagnosis) (AUC = 0.74) (95% confidence interval Cl, 67.3.80.4) (73,4% sensitivity, 67.4% specifity) (Figure 1).

Therefore, we considered a CONUT score of ≥ 2 as high and a score of < 2 as low. There was a statistically significant difference for the age (< 65 and ≥ 65 years) parameter between patients with a CONUT score >2 and those without (p = 0.002). There was a statistically significant difference between the patients with high (≥ 2) and low (< 2) CONUT scores and PS, LDH, bone marrow involvement, extranodal disease, high risk NCCN-IPI and stage IIIA–IVB (p = 0.001) (Table 3).

**Table 5:** Multivariate analysis for overall survival and progression free survival.

	Overall Survival			Progression Free Survival		
	HR	95%Cl	p	HR	95%Cl	p
Age ≥ 65	1.80	1.06–3.05	0.028	2.02	1.21–3.37	0.007
LDH	1.03	0.63–1.68	0.890	1.21	0.76–1.93	0.420
ECOG > 1	2.04	1.22–3.42	0.006	1.65	0.99–2.76	0.053
Bone marrow involvement	1.02	0.53–1.94	0.939	1.26	0.67–2.35	0.468
Extranodal Disease	0.73	0.39–1.36	0.335	0.61	0.34–1.12	0.113
Stage (IIIA-IVB)	2.75	1.51–4.99	0.001	2.42	1.36–4.31	0.002
IPI(low- high)	1.38	0.61–3.08	0.432	1.32	0.60–2.89	0.478
CONUT score	1.15	1.04–1.26	0.003	1.19	1.08–1.31	0.001

(AUC = 0.74) (95% confidence interval Cl, 67.3–80.4)

### 3.2. Overall survival and progression-free survival 

In the univariate analysis for OS, age ≥ 65 (HR 3.19, 95% Cl 2.03–5.01, p = 0.001), ECOG > 1 (HR 1.97, 95 Cl% 1.69–2.31, p = 0.001), bone marrow infiltration (HR 3.02, 95% Cl 2.05–4.98, p = 0.001), presence of extranodal involvement (HR 1.61, 95% Cl 1.07–2.43, p = 0.023), stage IIIA–IVB disease (HR 4.37, 95% Cl 2.86–6.69, p = 0.001), high IPI risk (HR 4.83, 95% Cl 3.17–7.36, p = 0.001) and CONUT scores (HR 1.23, 95% Cl 1.15–1.31, p = 0.001) were found to be statistically significant. In the univariate analysis for PFS, age ≥ 65 (HR 3.17, 95% Cl 2.04–4.92, p = 0.001), ECOG > 1 (HR 1.94, 95% Cl 1.65–2.27, p = 0.001), increased LDH level (HR 1.52, 95% Cl 1.02–2.28, p = 0.038), bone marrow infiltration (HR 3.04, 95% Cl 1.96–4.72, p = 0.001), stage IIIA–IVB disease (HR 4.07, 95% Cl 2.68–6.18, p = 0.001), high IPI risk (HR 4.52, 95% Cl 3–6.82, p = 0.001) and CONUT scores (HR 1.24, 95% Cl 1.15–1.33, p = 0.001) were found to be significant (Table 4).

**Table 3:** Comparing between the CONUT score low (< 2) and high (≥ 2) patients.

		Total(n = 266)	Conut < 2(n = 146)	Conut ≥ 2(n = 120)	p
Age	< 65	136 (51.1%)	87	49	p = 0.002
	≥ 65	130 (48.9%)	59	71	
Sex	Female	131 (49.3%)	67	64	p = 0.227
	Male	135 (50.7%)	79	56	
ECOG	< 1	158 (59.4%)	105	43	p = 0.001
	≥ 1	108 (40.6%)	41	67	
LDH	normal	136 (51.1%)	91	45	p = 0.001
	> normal	130 (48.9%)	55	75	
Bone marrow involvement	-	217 (81.6%)	137	80	p = 0.001
	+	49 (18.4%)	9	40	
Extranodal Disease	-	133 (50%)	93	40	p = 0.001
	+	133 (50%)	53	80	
Stage	IA-IIB	159 (59.8%)	114	45	p = 0.001
	IIIA-IVB	107 (40.2%)	32	75	
IPI (low,low intermediate)		167 (62.8%)	129	38	p = 0.001
IPI (high intermediate,high)		99 (37.2%)	17	82	

**Figure 2:**
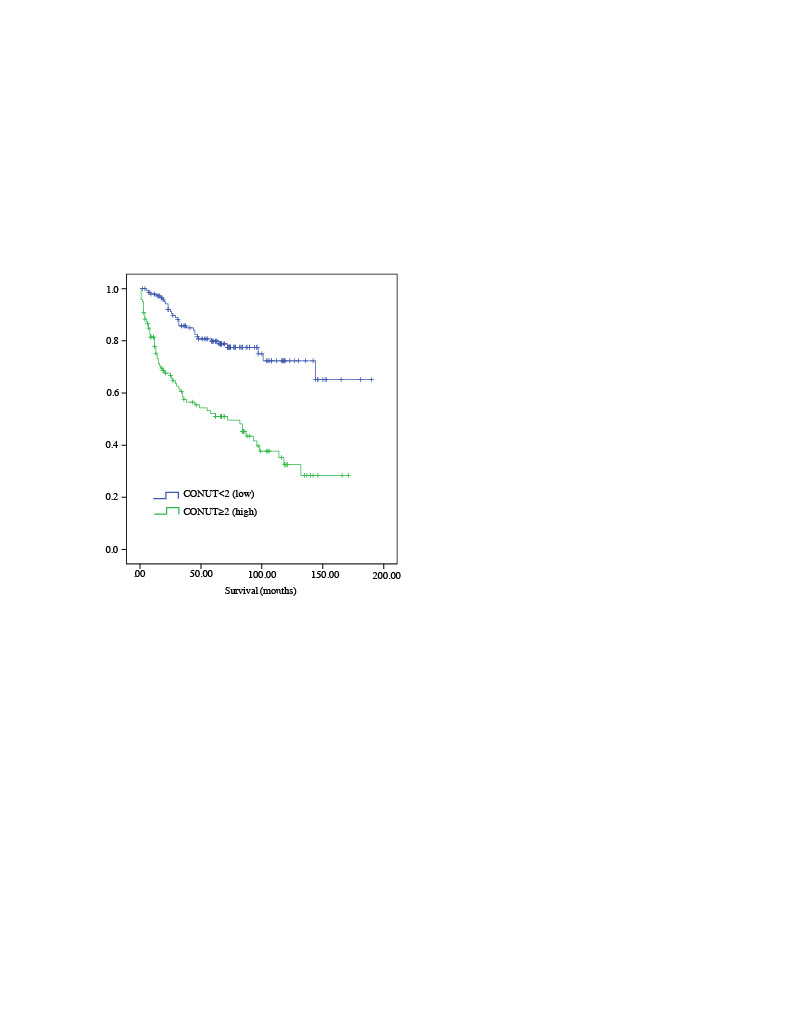
Comparing overall survival (OS) and CONUT score (< 2) and (≥ 2).

**Figure 3:**
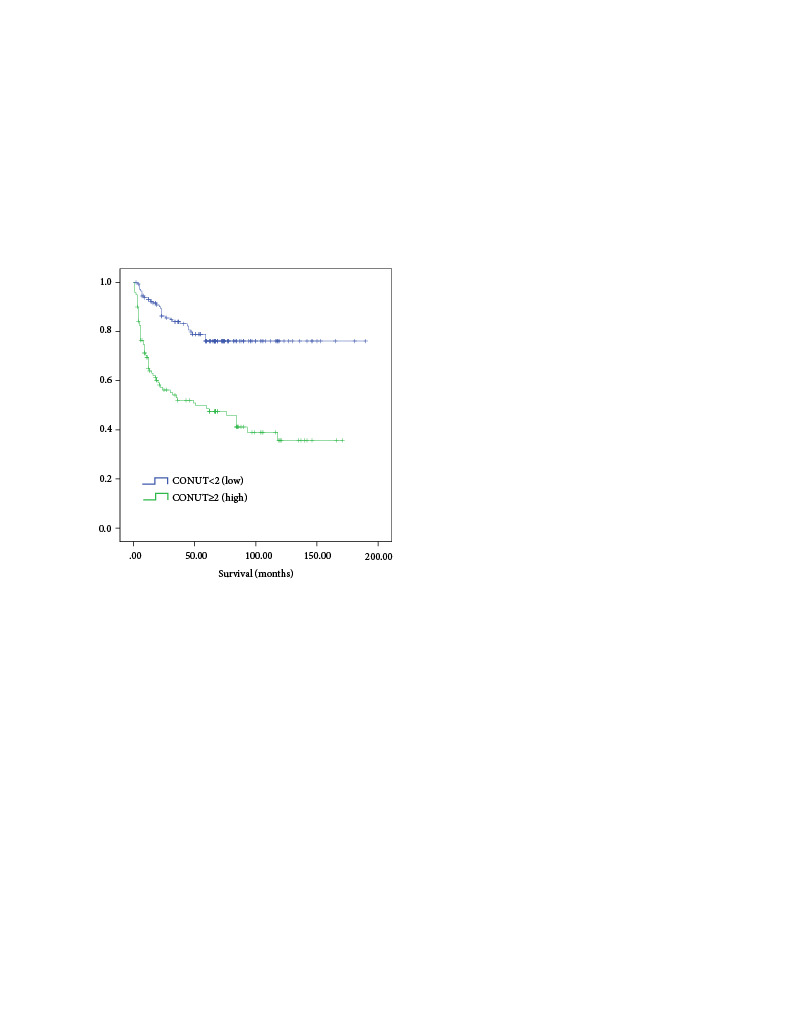
Comparing progression free survival (PFS) and CONUT score (< 2) and (≥ 2).

In the multivariate analysis for OS, age ≥ 65 (HR 1.80, 95% Cl 1.06–3.05, p = 0.028), ECOG >1 (HR 2.04, 95% Cl 1.22–3.42, p = 0.006), stage IIIA–IVB disease (HR 2.75, 95% Cl 1.51–4.99, p = 0.001) and the CONUT score (HR 1.15, 95% Cl 1.04–1.26, p = 0.003) were found to be statistically significant. In the multivariate analysis for PFS, age ≥ 65 (HR 2.02, 95% Cl 1.21–3.37, p = 0.007) and stage IIIA–IVB disease (HR 2.42, 95% Cl 1.36–4.31, p = 0.002) and CONUT score (HR 1.19, 95% Cl 1.08–1.31, p = 0.001) were found to be significant (Table 5). There was a negative correlation between the CONUT score and OS (r = −0.303, p = 0.001) and PFS (r = −0.329, p = 0.001). As the CONUT score increases, OS and PFS decrease. In addition, there was significant difference between the patients with high (≥ 2) and low (< 2) CONUT scores in terms of OS and PFS. Five-year OS and PFS in patients with high CONUT scores were 52.1% and 49.7%, respectively. Five-year OS and PFS in patients with low CONUT scores were 79.8% and 75.6%, respectively (p < 0.001) (Figures 2,3). 

**Table 4:** Univariate analysis for overall survival and progression free survival.

	Overall Survival			Progression Free Survival		
	HR	95%Cl	p	HR	95%Cl	p
Age ≥ 65	3.19	2.03–5.01	0.001	3.17	2.04–4.92	0.001
Sex	0.79	0.53–1.18	0.250	0.84	0.56–1.25	0.384
ECOG > 1	1.97	1.69–2.31	0.001	1.94	1.65–2.27	0.001
LDH	1.49	0.99–2.25	0.053	1.52	1.02–2.28	0.038
Bone marrow involvement	3.2	2.05–4.98	0.001	3.04	1.96–4.72	0.001
Extranodal Disease	1.61	1.07–2.43	0.023	1.42	0.95–2.12	0.085
Stage (IIIA-IVB)	4.37	2.86–6.69	0.001	4.07	2.68–6.18	0.001
IPI(low- high)	4.83	3.17–7.36	0.001	4.52	3– 6.82	0.001
CONUT score	1.23	1.15–1.31	0.001	1.24	1.15–1.33	0.001

## 4. Discussion

CONUT score is for scoring to assess the nutritional and immune status. The CONUT score has been shown to be associated with disease progression and mortality in cancer patients. Poor nutritional status both increases chemotherapy-induced toxicity and negatively affects the response to chemotherapy [8,9]. Our study found that OS and PFS decreased in patients with high CONUT scores. We have shown that a high CONUT score is an independent, strong prognostic index in patients with DLBCL. 

Malnutrition is observed in 30%–85% of patients with advanced stage cancer. There are studies regarding loss of weight, sarcopenia, low body mass index and low serum albumin to define malnutrition in patients with malignancy [10,11]. Albumin is the most abundant plasma protein in the blood and synthesised in the liver. Serum albumin is known to be associated with prognosis in various cancer types. Lymphocytes include CD4, CD8 T cells, natural killer cells, gamma–delta T cells and B cells. Decreased lymphocyte count is associated with impaired immunity, which causes the progression of the tumour [9,12]. CONUT score is calculated by measuring serum albumin, lymphocyte count and total cholesterol levels. Recently, the CONUT score, which is a nutritional index, has been used for the definition of malnutrition. It has been reported that the CONUT score is a prognostic factor that has an effect on survival in colorectal, gastric, oesophageal, hepatocellular, cholangiocarcinoma and lung cancers [13–19]. 

There are a limited number of studies showing the effect of CONUT score in haematological malignancies. The studies by Okamoto et al. and Ureshino et al. found that the CONUT score is a prognostic factor in multiple myeloma and T cell leukaemia/lymphoma, respectively [5,8]. There are only studies by Nagata et al. and Matsukawa et al. on the prognostic significance of CONUT score in patients with DLBCL [20,21]. We also evaluated the difference of CONUT score with prognostic factors. There was a significant difference between high CONUT scores and older age, worsened PS, increased LDH, bone marrow involvement and extranodal disease, stage IIIA–IVB and high risk NCCN-IPI (high-intermediate, high). We found the CONUT score higher in patients with progressive disease than in those without. Our study found a negative correlation between CONUT score and OS and PFS. We found that as the CONUT score increased, OS and PFS decreased. In addition, there was a significant difference between patients with high (≥ 2) or low (< 2) CONUT scores in terms of 5-year OS and PFS. Our results were similar to those in the studies by Nagata et al. and Matsukawa et al. [20,21]. We showed that the CONUT score has an effect on survival regardless of age and stage in patients with DLBCL. Our study showed that the CONUT score in patients with DLBCL is a strong index of poor prognosis. 

## 5. Conclusion

We found that the high CONUT score is a useful indicator of survival in DLBCL patients. The CONUT score is an easy-to-calculate scoring method that can be performed during routine blood draws from DLBCL patients. Our study is one of the limited number of studies showing the relationship between the CONUT score and prognostic factors in DLBCL patients.

Our study shows that the CONUT score is an independent, strong prognostic index in patients with DLBCL. However, there is a need for prospective studies with a larger sample size for long-term reliability and acceptability. 

### 5.1. Limitation

The present study was designed as a retrospective and single centre study. In our study, the patients’ calorie intake, nutritional status and body mass index at the time of diagnosis were not specified as they were not recorded. The cut-off value for the CONUT score was determined based on the patients’ remission, refractory or relapse status. The pathological phenotype of patients treated with DA-REPOCH chemotherapy was non-germinal. Our centre has been able to differentiate between germinal and non-germinal types since 2017. This differentiation could not be performed in patients with a diagnosis date before 2017. 

### Ethics approval

The present study was approved by the Institutional Ethics Review Board of Pamukkale University Faculty of Medicine (date: 10.06.2020, no: 60116787-020/34148). All procedures performed in studies involving human participants were in accordance with the ethical standards of the institutional and/or national research committees and the Declaration of Helsinki. 

## Informed consent

We did not obtain informed consent from the patients because of the retrospective design of the data collection.
